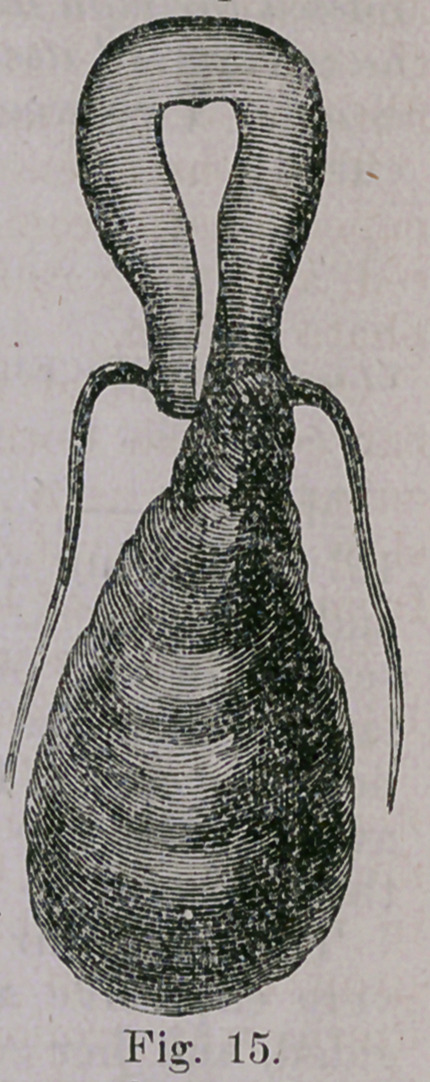# Clinical Notes on the Electric Cautery in Uterine Surgery

**Published:** 1873-02

**Authors:** J. Byrne

**Affiliations:** Surgeon-in-Chief to St. Mary’s Hospital for Diseases of Women; Clinical Professor of Uterine Surgery to Long Island Medical College, etc.


### Miscellaneous.

### Clinical Notes on the Electric Cautery in Uterine Surgery.

BY J. BYRNE, M. D.,

*Surgeon-in-Chief to St. Mary's Hospital for Diseases of Women; Clinical Professor of Uterine Surgery to Long Island Medical College, etc.*

### CASE XIII.

INTESTITIAL FIBROID.

Miss ———, aged 22, sought advice on account of menorrhagia, in March, 1869, which had existed for about 12 months previously. At this time her friends stated that she seemed to be increasing in size, and that a hard swelling had been noticed towards the lower and right side of her abdomen, but no examination was made until august of the same year. At this period a large globular and firm tumor was found occupying the right iliac fossa, and a digital examination per vaginum discovered the os uteri dilated to its utmost capacity and this same body presenting. The margin of the open cervix was traceable only to the extent of one-half its circumference, the remaining or right half being continuous with the intra-uterine tumor. Menorrhagia was very profuse, and each catamenial period was likened to a severe and prolonged labor, being attended with violent expulsive pains of an intermittent character. In September, 1869, an attempt to draw down the tumor was made with a view of removing it, but its sessile character was such as to render the effort impracticable.

In December, 1869, Professor Barker saw the case in consultation with her attending physician, Dr. Schapps, diagnosed a recurrent fibroid, and discouraged any attempts for its removal. Up to November, 1871, the tumor continued to increase in size upward as well as within the vagina, and extended from two inches above and to the right of the umbilcus down to the vulva. The pelvic cavity was now completely filled up with the firm, irregularly lobulated mass; defecation was seriously impeded, and the frequent use of a catheter was called for to empty the bladder, which could only be entered by a long flexible one and with much difficulty. Menorrhagia was not so excessive as formerly, but the violent expulsive pains already referred to still recurred with each catamenial period. She was now in a most deplorable condition from long suffering and loss of blood, and at this period in her history I saw her for the first time at the request of Dr. Schapps. By placing the patient on her side and drawing back the perinæum, a sound could be passed into the uterine cavity, and plainly felt through the abdominal wall above and to the left of the umbilicus, and the depth measured at least ten inches. The vaginal mass was firm and elastic to the touch, and numerous large-sized blood-vessels were observed ramifying on its surface. An attempt to remove this intra-vaginal part by galvano-cautery was now proposed and consented to. The operation, which took place November 16, 1871, may be described as follows: A strong semi-circular needle, seven inches in length exclusive of handle, with eye 3-8 of an inch from point, and carrying a heavy thread, was made to penetrate the tumor posteriorly as high up as could be reached, and was pushed forward until the point could be felt behind the pubic arch, provision being made to protect the urethra from injury. A slight additional force enabled me to reach the thread by means of a tenaculum, and the needle was withdrawn, while one end of the thread was brought down anteriorily. A

FIG. 11.

strong platina wire being attached to the cord, was next drawn through and made to take the place of the latter. At this stage some trifling hemorrhage was observed. A connection was now made to the battery, and by very slow traction, occupying at least fifteen minutes, the tumor was split down longitudinally, and thus divided into two nearly equal halves and without loss of blood. The left half of the mass was now looped, and its removal effected with comparatively little difficulty. An effort was next made to dispose of the remaining portion by the same process, but after repeated trials this method was found impracticable, principally on account of its more irregular and conical shape. Recourse was now had to the cautery knife, when the whole was removed piecemeal, and all irregular projections within the pelvic cavity being trimmed off, the operation, which lasted two hours and a quarter, was thus completed.

The patient's recovery from the effects of the operation was rapid, and unattended with the slightest inflammatory symptoms or irritative fever. Relief from the more distressing symptoms was complete; her appetite and strength rapidly returned, and though no attempt at spontaneous enucleation of the upper segment of the tumor took place, an occurrence faintly hoped for, yet her general health continued to improve, and for a period of over six months her life was one of comparative comfort.

In the early part of June, however, Dr. Schapps informed me that, though the abdominal part of the tumor had not apparently increased, the pelvic growth had to some extent reappeared, and the menstrual expulsive pains returned with much severity.

On the 15th of last August I was urgently requested to see her, on account of great difficulty having been found by Dr. Colt, Dr. Schapps being then in Europe, in emptying the bladder by catheter, following an unusally severe and long continued attack of her periodical expulsive pains. Her suffering was described by her mother as equal to a severe labor, and she was hourly expecting a return of the same agony, which, in her now amaciated and anæmic condition, it was thought impossible she could survive. The tumor was to be seen bulging out between the vulva, and a flexible catheter was passed into the bladder with much difficulty. On the night of the 17th she was seized with the dreaded pains, and during one violent paroxvsm a large part of the tumor was forced

FIG. 12

through the vaginal outlet, lacerating in its passage the perinæum and one side of the vulva.

Its size, shape, and general appearance will be understood by reference to the foregoing illustration, and the dotted lines indicate the form and position of its upper pelvic and abdominal portion. The protruding part measured 9 inches in length, and from 14 to 15 inches in circumference. For a space of two or three inches from its lower end sphacelated discoloration was observed, and the odor of decomposition was strongly marked. Numerous large blood vessels were seen ramifying on its surface, the upper two-thirds of which was of a deep red color, from interruption in its circulation; while in consistence it presented the firm character of an ordinary fibroid.

THE OPERATION.

The patient being anæsthetized, powerful traction was made below, while steady pressure was kept up on the supra-pelvic extremity of the tumor; but after continued efforts it was found impossible to bring it down more than one inch beyond the position it had already attained, owing in part to its connections within, but principally on account of its larger dimensions above. A double ligature of strong whip-cord was now passed from behind forward through the centre of the tumor, immediately outside the perineal commissure, steady traction being all the time kept up, and the mass ligated in the usual manner, the principal object being to insure full control of the stump during and after excision. As the vascular appearance of the parts forbade the use of any ordinary sized platina wire, a piece six inches in length of No. 16 (Stubb's gauge) was fastened by binding screws between the two conducting cords of the battery, and curved so as to adapt itself to the contour of the tumor. This was now applied, *while cold*, to the under surface, half an inch below the ligature; and all being in readiness, the battery was next immersed, and the heated wire slowly carried around the tumor, as in circular amputation, thus effecting a deep fissure, and completely sealing up the superficial vessels. The battery was now raised and the wound examined, but no disposition to hemorrhage was observable. The wire was next applied to the under surface of the tumor as in the first instance, the battery reimmersed, and by a slow and steady see-saw movement the whole mass was cut through. Though the ligatures had by this time become quite loose from traction, there was no bleeding from the stump; nevertheless, in order to guard against secondary hemorrhage, the whole surface was well seared over a second time, and the dome-shaped cauterizer pressed into every suspicious point.

The stump was then returned within the vagina, and an anodyne suppository of belladonna and morphine ordered, but no dressing of the wound was used or deemed necessary.

As space will not permit a detailed record of her progress after the operation, I will merely add that, though suffering from two extensive bed-sores, she improved rapidly and without the slightest symptom of local inflammation or irritative fever. The ligatures were allowed to remain for three or four weeks, with the hope of effecting some reduction in the upper tumor by drainage; but their presence giving rise to a good deal of annoyance, and for other obvious reasons, they were taken away.*

### CASE XIV.

### CASE OF SESSILE INTRA-UTERINE FIBRIOD.

Mrs. D., aged 30, widow, has had five children and one miscarriage. Menstruation was always regular up to two years and a half ago, when her periods commenced to be prolonged and the flow excessive. She states that she has been under observation at Bellevue Hospital for about three months previous to her admission into St. Mary's, which was on the 15th of April, 1872. Her metrorrhagia had been for some time past almost continual, and as she was much reduced from loss of blood, it was deemed best to prescribe rest, nourishments and local astringents, before submitting her to the ordeal of a thorough examination. On the 1st of May, her condition having greatly improved, an investigation was made with a view to diagnosis and with the following result: Above the pubis and a little to the left was noticed a firm globular tumor, in size about that of a four months' pregnant uterus, somewhat tender to the touch, and slightly moveable from side to side. A digital examination revealed the presence of an intra-uterine tumor presenting within the os, which was soft and dilated to the extent of a silver dollar. The growth resembled an ordinary fibrous polypus, and it appeared to be free and detached from the uterine wall as far as the finger could reach, but owing to its large size (being about that of a human heart, which in shape and consistence it resembled), and as in its upper half it seemed to fill the entire cavity, the true character of its connection could not then be made out. I had not the good luck at this time to be made acquainted with the simple and ingenious device of Prof. Thomas, by the aid of which I have no doubt I might have been able to estimate the extent of its attachment.

The case was therefore diagnosed as one of intra-uterine fibrous polypus, and most probably pediculated. It should also be stated that manipulation with the sound failed to give any clear idea of the nature of its attachment.

On the 4th of May, the patient being anæsthetized, the cautery loop was passed into the uterine cavity and over the tumor; but as the latter was now found to be much less movable than

* A third operation has since taken place, and will be described before the close of this paper.

at first supposed, this step in the operation was attended with the utmost difficulty. I soon noticed that the wire could not possibly be made to embrace the outgrowth sufficiently far up to remove it entire, and now for the first time the real character of its attachment admitted of little doubt.†

A strong vulsellum forceps, being once more carefully passed through the loop and into the cavity, was opened, and the apex of the tumor laid hold of. Firm traction to the extent of partially inverting the uterus was then steadily maintained, while the loop was passed up as far as possible and tightened. The conductors were then attached and the battery immersed, when by a slow movement of the screw in the loop-handle the part embraced was cut thro' and removed. Space being now afforded for the introduction of two fingers, it was found that but little more than one-half of the tumor had been taken away. A repetition of the proceedings just described resulted in the removal of the remaining half, the surface from which it was taken being slightly elevated at its circumference, and seemingly about 2½ inches in diameter.

No blood was lost during the operation beyond what would necessarily come from handling the parts, nor was there any secondary hemorrhage. The uterus was injected daily with a weak solution of carbolic acid and vinegar, and the after-treatment in other respects consisted of beef-tea, milk punch and tonics, with an occasional anodyne suppository. Two weeks after the operation there was a trifling bloody discharge when the uterine cavity was explored by a polypus-forceps, and a portion of slough removed. A strong solution of iodine was then freely applied and

† The attachment of the tumor is not quite correctly represented in the above sketch, the upper portion being less spread out and proportionately narrower than the actual condition observed would warrant.

no further bleeding occurred. On the 30th of May, twenty-six days after the operation, the cavity of the uterus measured a little over three inches, and as the patient seemed to be daily improving, she was pronounced out of all danger. She left hospital on the 3d of June.

### CASE XV.

### FIBROUS POLYPUS OF THE UTERUS.

Kate ———, aged forty-five, unmarried, had always enjoyed good health and menstruation regularly up to June, 1870. About this date she says the intervals between her courses began to be prolonged and the flow scanty, but that towards the end of December she was taken with "flooding," which lasted two weeks. Throughout the year 1871 she had attacks of metrorrhagia, sometimes lasting for ten and even fifteen days, and for the cure of which she stated she had taken "a power of medicine." She noticed some increase in the size of her abdomen, but it did not engage her attention to any extent; and on the 30th of December, 1871, she was seized with severe hypogastric pain and "bearing down," when a large tumor made its appearance outside the vulva. Dr. J. P. Dwyer was now called to see her, diagnosed a fibrous polypus, and recommended her to be sent to St. Mary's hospital for operatian.

14

On examination the tumor was found to be firm and lobulated, and in size about twice that of a closed hand. Its pedicle, which measured about four inches in length, was round, and about one inch in diameter at its smallest part, which appeared to be midway between the tumor and its uterine attachment. Affixed to the pedicle, about an inch and a half from the tumor, was a small pediculate fibroid outgrowth.

On attempting to pass a sound into the uterus, which appeared fully dilated, it was found impossible to carry it beyond one inch anteriorily and less than half that distance either behind or in a lateral direction. A finger passed into the rectum came in contact with a firm body as far as could be touched, and conjoined pressure over the pubes failed to convey any very definite idea as to the form or position of the fundus. Nevertheless, partial inversion of the uterus was diagnosed, and accordingly, *instead of proceeding to sever the pedicle near what seemed to be its uterine insertion, the point selected was half an inch above the little secondary outgrowth. When the heated wire had passed through and the tumor was removed, the uterus was found to have reverted itself and the cavity measured over three inches in depth.* Two weeks after the operation the patient was discharged cured.

:o:

### CASE XVI.

### LARGE FIBRO-CELLULAR POLYPUS OF THE CERVIX; FIRST NOTICED FIVE DAYS AFTER PARTURITION.

Mrs. M——, aged 28, was delivered of her third child April 6th, 1870. During gestation nothing occurred to excite her suspicions, and her general condition was in no way different from that observed in two previous pregnancies. In this third labor, which lasted but a few hours, she was attended by a midwife, and no difficulty occurred further than that the after-birth was slow to come away. Yet she was sure no undue traction had been made on the cord.

Three or four hours after delivery she was seized with very severe expulsive after-pains, which lasted for three days, then subsided, and her condition for the following two days was, on the whole, comfortable.

On the fifth day, being without a nurse, and having no one to care for her children, she ventured to get up and walk about; but no sooner had she done so than a large substance, which she thought was her womb, protruded from the vagina. She immediately returned to bed, and so remained until I was requested to see her, which was on the 14th (eight days after confinement). During these three days there was a constant passive hemorrhage, and she appeared very weak and anæmic; but she complained of no pain, and the greater part of the tumor had retreated within the pelvic cavity soon after assuming the recumbent position. In shape it was ovoid, or rather pyriform, about the size of a uterus at from three to four month's gestation, and of firm consistence, except at its lower surface, where it yielded readily to pressure from below upward, but immediately recovered its convexity on the pressure being removed, thus giving a very distinct impression of its being hollow. Several abraded spots were observed on its sides and inferior surface, from which blood oozed, and the whole was of a deep flesh color.

In accordance with my advice, she was brought to St. Mary's Hospital April 16th, 1870, when a careful examination was made with the hope of deciding as to whether this was really a case of inversion of the uterus or a polypus. On introducig two fingers within the vagina and making traction on the prolapsed mass with the other hand, it was found that there was no cervical rim, but, on the contrary, the vaginal surfaces and that of the tumor were continuous, except at one small spot anteriorly, which was depressed. Here an effort was made to introduce a probe or sound, but unavailing. By examination per rectum and pressure above pubes, I failed to satisfy myself of the presence of a uterus above, and for the time being desisted from further efforts at diagnosis. At this juncture, the case being one of unusual interest, I requested Drs. Thomas, Noeggerath, and James L. Brown to see her with me. The same steps towards forming a diagnosis were again resorted to, and after repeated efforts Dr. Thomas managed to get a probe into the cavity of the uterus from the bottom of the little concavity in front, and thus all doubts as to the position of that organ and the character of the tumor were at an end. It is but proper to state, however, that before the cavity of the uterus was reached all present felt certain of having detected, by bi-manual examination, a body which it did not seem possible could be any other than the uterus. Nevertheless, had every attempt to reach the cavity of the uterus failed, and no other evidence of its existence above been found than that afforded by the rectal and supra-public touch, the true nature of the case must still have remained doubtful; because, supposing this to have been a case of inversion, it is very easy to imagine how a subperitoneal fibroid might have swung into the position vacated by the inverted uterus, and thus deceive the very best diagnostician.

Fig. 15.

Again, though, as Dr. Thomas observes,* the presence of a body in the uterine region may warrant a more or less forcible introduction of a probe when, owing to the agglutination of tissues by inflammatory action, the aperture may have been closed, it should not be forgotten that under such circumstances but a small amount of force would be needed to effect a passage into cellular tissue or elsewhere in this immediate neighborhood.

At all events, this case, if not unique, is so interesting and instructive that no apology is needed for occupying so much space with its history.

The operation for the removal of this polypus was also no less profitable than interesting, because, in addition to errors committed in operating, and, of course, carefully avoided ever after, all my subsequent experiments towards devising a more powerful and yet portable battery than had been generally used heretofore, were prompted by what was observed on this occasion. In the first place, though the battery employed was one of huge dimensions,

* Diseases of Women, 3d edition, p. 412.

the thickness of the wire which it was capable of heating was quite insufficient to thoroughly cauterize the tissues in its passage through the pedicle; secondly, I contracted the loop too rapidly; and lastly, to make the matter still worse, traction was made on the tumor, so that, like ripping a seam in cloth, while some of the fibres were cut, many were barely touched with the heated wire.

The consequence of all this was, that my patient narrowly escaped death from hemorrhage. One large artery had to be ligated, and the vagina was tamponed with oakum soaked in persulphate of iron.

On account of this latter objectionable application, of which I can conceive nothing more filthy and abominable under all circumstances as a uterine or vaginal styptic, the cut surface was slow to heal, yet the patient was discharged well within a month from the date of her admission.

She has since given birth to her fourth child, and is in the enjoyment of perfect health at present.

This case is suggestive of many pathological theories and speculations; but the limits of this paper will not permit me to say more than that I believe the formation of this polypus commenced in the cervical canal before or soon after conception; that its growth took an upward direction; and, as a development of the uterus was proportionately greater and more rapid than that of the tumor, there was thus ample room afforded for its safe accommodation during gestation.

## Figures and Tables

**Fig. 11. f1:**
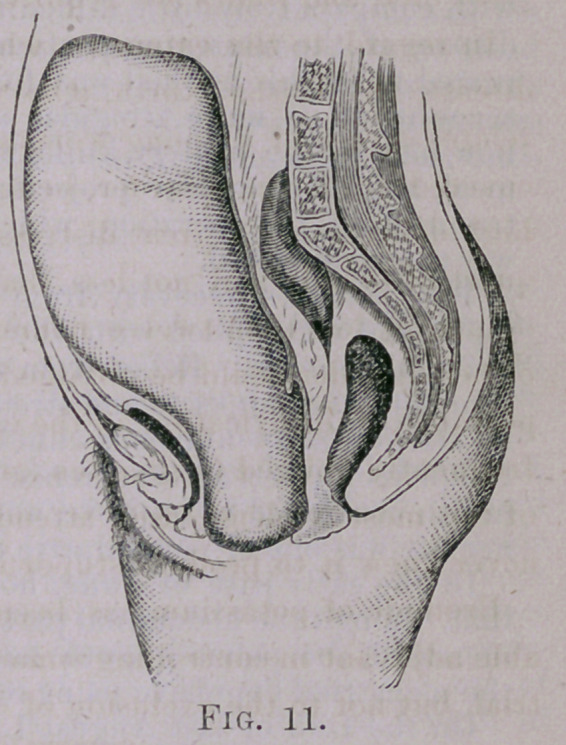


**Fig. 12. f2:**
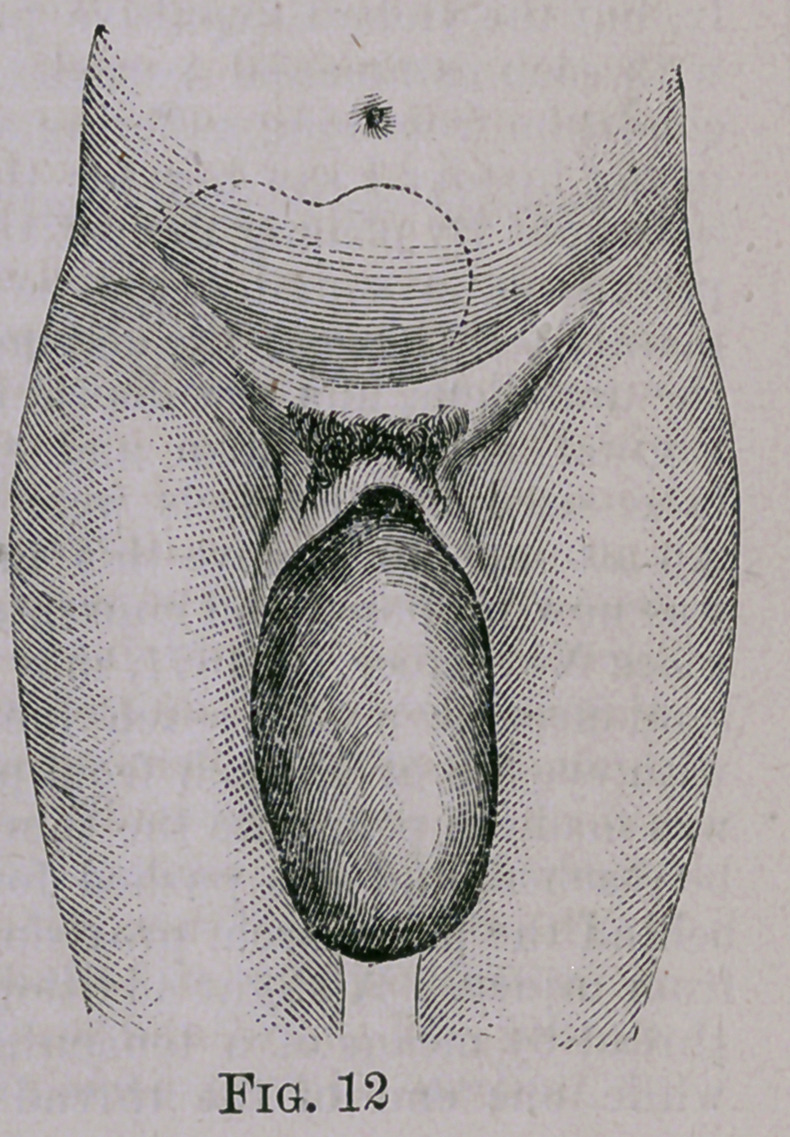


**Figure f3:**
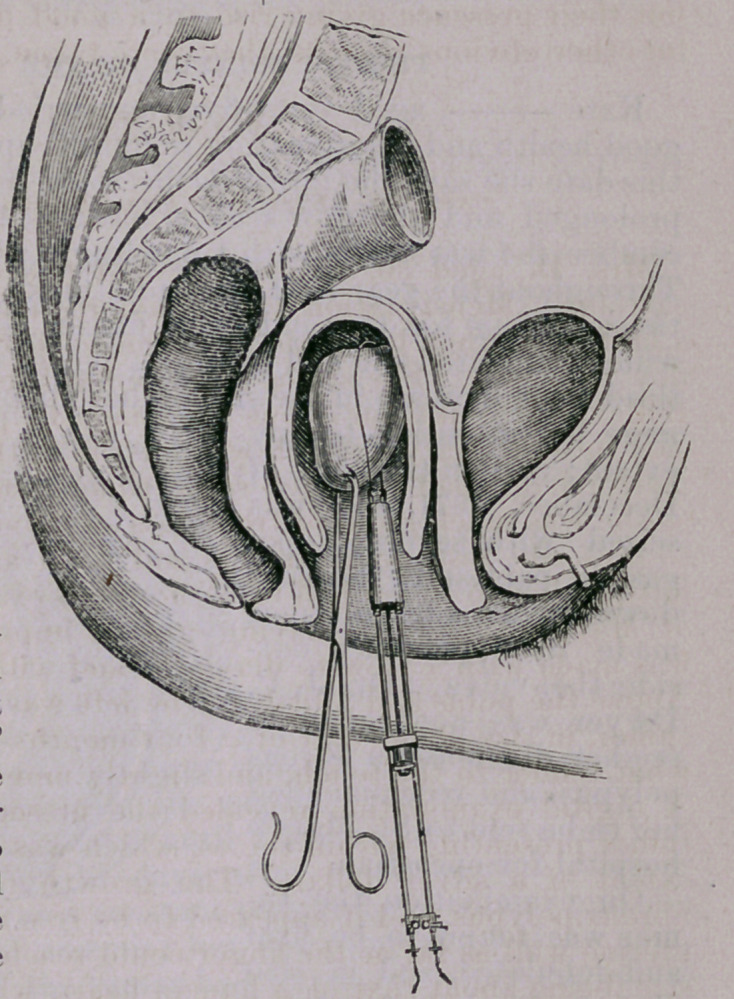


**14 f4:**
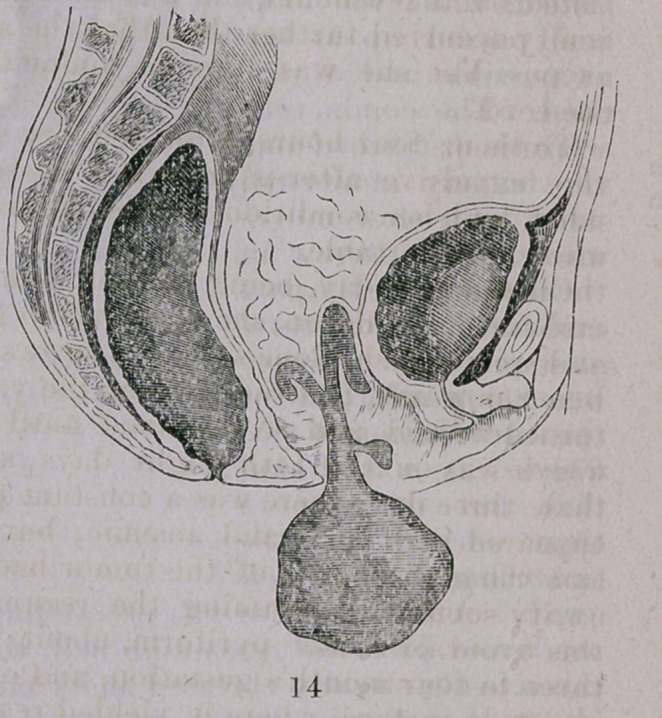


**Fig. 15. f5:**